# Transcription Factor Oct1 Is a Somatic and Cancer Stem Cell Determinant

**DOI:** 10.1371/journal.pgen.1003048

**Published:** 2012-11-08

**Authors:** Jessica Maddox, Arvind Shakya, Samuel South, Dawne Shelton, Jared N. Andersen, Stephanie Chidester, Jinsuk Kang, Keith M. Gligorich, David A. Jones, Gerald J. Spangrude, Bryan E. Welm, Dean Tantin

**Affiliations:** 1 Department of Pathology, University of Utah School of Medicine, Salt Lake City, Utah, United States of America; 2 Department of Oncological Sciences, Huntsman Cancer Institute, University of Utah School of Medicine, Salt Lake City, Utah, United States of America; 3 Department of Surgery, Huntsman Cancer Institute, University of Utah School of Medicine, Salt Lake City, Utah, United States of America; University of Washington, United States of America

## Abstract

Defining master transcription factors governing somatic and cancer stem cell identity is an important goal. Here we show that the Oct4 paralog Oct1, a transcription factor implicated in stress responses, metabolic control, and poised transcription states, regulates normal and pathologic stem cell function. Oct1^HI^ cells in the colon and small intestine co-express known stem cell markers. In primary malignant tissue, high Oct1 protein but not mRNA levels strongly correlate with the frequency of CD24^LO^CD44^HI^ cancer-initiating cells. Reducing Oct1 expression via RNAi reduces the proportion of ALDH^HI^ and dye efflux^HI^ cells, and increasing Oct1 increases the proportion of ALDH^HI^ cells. Normal ALDH^HI^ cells harbor elevated Oct1 protein but not mRNA levels. Functionally, we show that Oct1 promotes tumor engraftment frequency and promotes hematopoietic stem cell engraftment potential in competitive and serial transplants. In addition to previously described Oct1 transcriptional targets, we identify four Oct1 targets associated with the stem cell phenotype. Cumulatively, the data indicate that Oct1 regulates normal and cancer stem cell function.

## Author Summary

Understanding the mechanisms that control stem cell function is a fundamental prerequisite both for the full application of stem cells to regenerative medicine and for a full understanding of the relationship between stem cells and cancer. In this study we show that a transcription factor known as Oct1 is a central regulator of normal and cancer stem cell function. We show that high Oct1 levels are associated with stem cells in multiple normal and malignant settings. Altering Oct1 expression, up or down, correspondingly alters multiple stem cell parameters, as well as stem cell function. We highlight known and identify new target genes Oct1 binds to that are consistent with a role in stem cell function.

## Introduction

Mammalian somatic stem cells have been identified in blood, lung, intestine, breast, epidermis and other tissues [1]–[6]. Cancer stem cells (or cancer-initiating cells) have been defined in a variety of developmentally heterogeneous neoplasms [7]–[11]. Two functional properties are consistently used to define both normal stem cells and cancer stem cells (CSCs): the ability to self-renew and the ability to generate progeny cells with more differentiated phenotypes [12]. Because CSCs may have metastatic ability, and are thought to be chemo- and radio-resistant and thus provide a reservoir for replenishing tumor mass [13]–[15], there is interest in identifying cellular activities that regulate CSC populations. Similarly, the central role of somatic stem cells in maintaining tissue homeostasis places priority on identifying cellular activities governing their function. Wnt-, Notch- and Hedgehog-mediated signaling contributes to the maintenance of certain adult somatic and CSC populations [16]–[18]. Identifying additional regulators would allow for robust stem cell identification and provide possible therapeutic targets.

The Oct1 transcription factor is widely expressed in adult tissues. It is related to Oct4, a regulator of embryonic stem (ES) cell pluripotency, and has similar in vitro DNA binding specificity [19]. Oct1 enforces poised transcriptional states [20] and promotes a glycolytic metabolic profile associated with dampened mitochondrial function and reactive oxygen species (ROS) levels [21]. This metabolic state is emblematic of both tumor cells and stem cells [13], [22]–[25]. Loss of Oct1 has little impact on cell growth and viability in culture, or on immortalization by serial passage, but antagonizes oncogenic transformation *in vitro* and tumorigenicity *in vivo* [21]. Oct1 also promotes resistance to genotoxic and oxidative stresses, a phenotype observed in stem cells [26]. Oct1 message levels are increased in some forms of gastric cancer [27], however consistent changes in Oct1 gene expression have not been noted for most malignancies. Oct1 protein levels and post-translational modification states in stem cell compartments and malignancy have not been studied.

Here we show that Oct1 controls multiple stem cell phenotypes in normal and tumor cells. In epithelial cells, strong Oct1 protein expression spatially correlates with stem cell niches and high levels of ALDH1, Lrig1 and Lgr5, known stem cell markers. Elevated Oct1 protein expression also correlates with elevated ALDH^HI^ and CD24^LO^CD44^HI^ stem cell-like populations in tumor cell lines and primary breast cancer samples, respectively. In contrast, the correlation with Oct1 mRNA expression is poor. Using ALDH and dye efflux activity as readouts, we demonstrate that Oct1 ablation selectively depletes stem-like populations in multiple human tumor cell lines. *Abcg2*, *Abcb1, Abcb4* and *Aldh1a1*, genes associated with dye efflux and ALDH activity, are direct Oct1 targets. We show that stable Oct1 knockdown in two different tumor cell lines reduces tumor-initiating frequency, while Oct1 ectopic expression increases tumor initiation. Finally, we show that Oct1 deficient fetal liver hematopoietic progenitors manifest engraftment defects in competitive and serial transplantation situations. The results indicate that Oct1 protein can be used as a stem cell marker, and that Oct1 is a normal and malignant stem cell determinant.

## Results

### Elevated Oct1 protein expression is associated with epithelial stem cells

**Figure 1:**
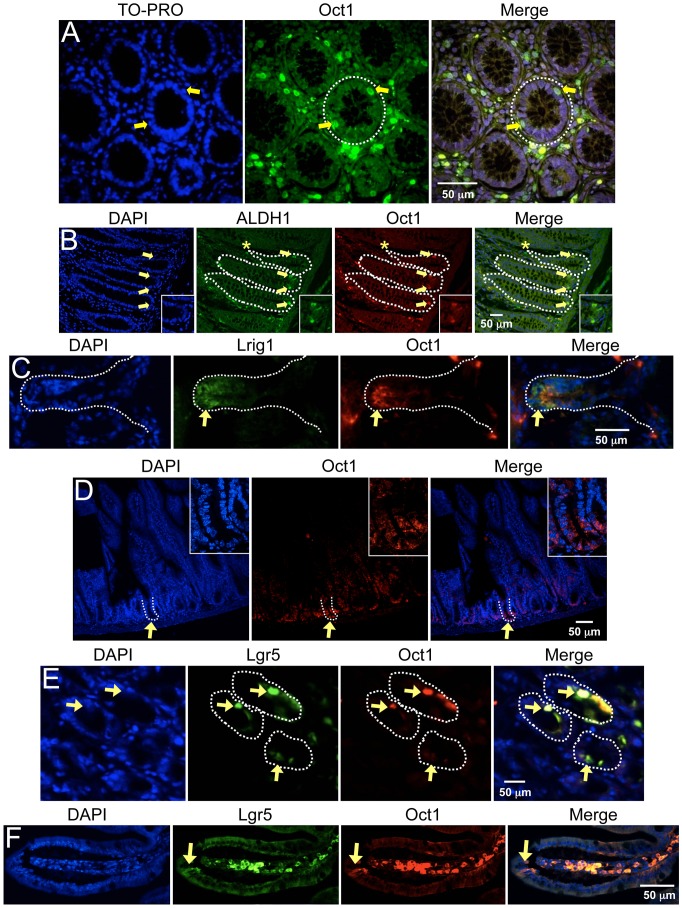
Association of Oct1 with normal somatic stem cells. A. IF images of cross-sections from grossly uninvolved colon margins of a male familial adenomatous polyposis patient. Frozen sections were stained with mouse anti-Oct1 antibodies (Millipore MAB5434) and co-stained with TO-PRO. Crypts are shown in cross-section. White dashed circle highlights a crypt. Arrows indicate cells staining strongly for Oct1. B. IF images of colon crypt sections from a normal male individual. Sections were stained with DAPI, and anti-Oct1 and anti-ALDH1a1 antibodies. Merged images are shown at right. White dashed lines highlight crypts. Examples of cells co-staining with Oct1 and ALDH1a1 are highlighted with yellow arrows. An example cell staining with ALDH1a1 only is highlighted with an asterisk. Inset at lower right-hand corner is a digital magnification of the central portion of the image. Sections were formalin-fixed and paraffin-embedded. C. Frozen mouse colon tissue sections were stained with DAPI, and anti-Lrig1 and anti-Oct1 antibodies. IF images of longitudinal sections are shown. White dashed lines highlight the crypt. D. IF images of mouse small intestine sections. Sections were stained with DAPI and anti-Oct1 antibodies. Merged images are shown at right. White dashed lines highlight a crypt. Inset at upper right-hand corner is a digital magnification of the central portion of the image. Sections were formalin-fixed and paraffin-embedded. E. IF images of cross-sectional duodenum sections from a normal C57BL/6 mouse. Frozen sections were stained with DAPI and anti-Oct1 and anti-Lgr5 antibodies. Merged images are shown at right. Examples of co-staining cells are highlighted with yellow arrows. White dashed lines highlights crypts. F. Longitudinal sections.

We examined Oct1 expression in frozen human colon sections using indirect immunofluorescence (IF). The Oct1 antibody (Millipore) did not cross-react to human Oct2, Oct4 or Oct6 in control experiments (Figure S1). IF revealed a sub-population of intensely staining Oct1^HI^ cells, one cell removed from the lumen but clearly within the basement membrane that defines the crypt (Figure 1A, arrows). Similar findings were made using immunohistochemistry (Figure S2). To examine the location and identity of these cells more closely, we used paraffin-embedded normal human colon sections with antibodies to Oct1 and ALDH1, which has been shown to mark somatic stem cells in multiple compartments, including the colon [9], [28], [29]. Oct1 and ALDH1 staining were widely detectable at low levels relative to controls lacking primary antibodies (not shown), consistent with the wide expression of these proteins. However, in a subset of cells, including within gut crypts, more intense co-staining was evident (Figure 1B). Merging the fields confirmed that cells at the crypt base stained strongly for both proteins (arrows). A few cells (not in the crypt base) displayed strong ALDH1 expression only (Figure 1B, asterisk). We identified multiple additional examples of staining with only one antibody (not shown), indicating that the results are not due to spectral overlap. We analyzed six independent tissue sections corresponding to 117 crypts, identifying 77% of cells with both Oct1^HI^ and ALDH1^HI^ expression. 19% of cells stained strongly with ALDH1 only and 4% with Oct1 only. These findings identified a high concordance between cells with high Oct1 and ALDH1 protein levels.

To extend these findings we also stained frozen mouse colon tissue sections with antibodies against Lrig1, which is highly expressed in stem cells at the crypt base [30], [31]. We identified general co-localization using anti-Oct1 and anti-Lrig1 antibodies, though the Oct1 staining was somewhat more restricted compared to Lrig1 (Figure 1C, arrows). Analysis of Lrig1-positive crypts indicated that 19/30 co-stained with high Oct1 expression. No crypts stained with Oct1 only but not Lrig1.

The small intestine crypt is one of the best-characterized stem cell niches. We examined Oct1 expression in formalin-fixed paraffin-embedded mouse small intestinal (duodenum) tissue. In this case we used different anti-Oct1 antibodies (from Bethyl). As with colon, IF revealed a sub-population of intense-staining Oct1^HI^ cells at the crypt base (Figure 1D, arrows). To examine these cells more closely, we performed co-localization studies using leucine-rich repeat-containing G protein-coupled receptor-5 (Lgr5). Lgr5 marks stem cells in the crypt [32], where it helps transduce Wnt signals [33], [34]. We used frozen normal mouse small intestine (duodenum) sections together with anti-Oct1 and anti-Lgr5 antibodies in IF. Oct1/Lgr5 co-staining was observed (Figure 1E and F, arrows). The signal was not due to nonspecific secondary antibody binding or autofluorescence, as removing either primary antibody eliminated signal only in the appropriate channel (Figure S3). Analysis of Oct1/Lgr5 co-localization using tissue from a green fluorescent protein-Lgr5 knock-in mouse [32] (a gift of H. Clevers) indicated that 27/30 Lgr5-positive crypts co-localized with Oct1 (90%). For Figure 1, longer exposure in the Oct1 channel and comparison to samples processed without the Oct1 antibody indicated that Oct1 was expressed in all cells, but much more strongly expressed in stem cells. Another protein, B lymphoma Moloney murine leukemia virus insertion region homolog-1 (Bmi1), is known to mark a different group of stem cells at the “+4” position in the small intestinal crypt [35]. Using duodenum from a tamoxifen-injected *Bmi1*-Cre-ER;*Rosa26*-Cre reporter mouse (a gift of M. Capecchi), we did not observe significant co-localization (0/27 Bmi1-positive crypts, data not shown). Cumulatively these data indicate that high Oct1 protein levels mark a specific population of normal stem cells in both colon and small intestine. The high Oct1 signal in stem cell compartments was not due to peculiarities with one particular antibody, autofluorescence or spectral overlap.

### Oct1 correlates with stem cell markers in primary malignant cells

**Figure 2:**
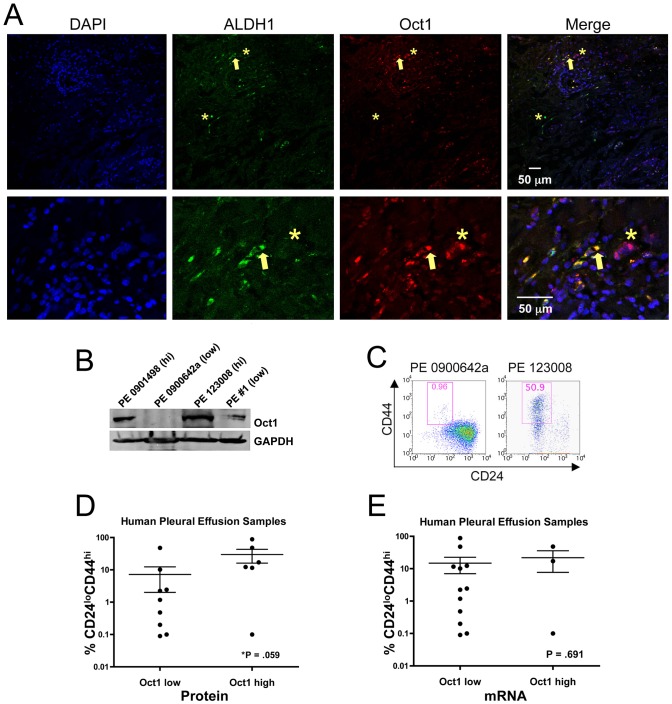
Oct1 protein levels correlate with a stem cell phenotype in primary human malignancy. A. IF images are shown. Frozen malignant breast carcinoma sections (non-familial stage IIIA node-positive infiltrating ductal carcinoma, ER^+^PR^+^HER2^−^) were stained with mouse anti-Oct1 antibodies (Millipore MAB5434) and rabbit anti-ALDH1 antibodies (Abcam ab52492). Arrow indicates example double-positive cell. Asterisks show examples of an Oct1^HI^ cell with low ALDH1 and ALDH1^HI^ with low Oct1. Detail is shown in images below. B. Western blot depicting Oct1 levels in a panel of malignant epithelial cells isolated from pleural effusions (human breast carcinoma lung metastases). GAPDH is shown as a loading control. C. Examples of the CD24/44 profile from pleural effusions with highest and lowest Oct1 protein levels. D. Correlation of Oct1 protein and CD24^LO^CD44^HI^ stem content in 15 individual patient samples (7 ER^+^PR^+^HER2^−^, 5 ER^−^PR^−^HER2^−^, 3 ER^−^PR^−^HER2^+^) collected from 14 different patients. One patient had tumor cells collected twice 22 months apart. Samples were placed into Oct1^LO^ and Oct1^HI^ categories based on Oct1 Western blot signal intensity relative to GAPDH staining. Cells were tested for percentage CD24^LO^CD44^HI^ stem cell content and plotted. *P*-value was calculated using the two-tailed student T-test. E. The same analysis for Oct1 mRNA as measured by qRT-PCR.

We performed Oct1/ALDH1 IF on malignant human breast carcinoma sections (estrogen receptor^POS^, progesterone receptor^POS^, Her2^NEG^). In addition to somatic stem cells, ALDH1 expression marks CSCs, including in breast and colon [9], [28], [36]. Dual staining was evident (Figure 2A, arrows) though again we observed examples of cells that stained with Oct1 and not ALDH1 and vice-versa (asterisks). These results indicate that Oct1 levels are elevated in a subset of breast cancer cells that also express high levels of ALDH1.

To corroborate these findings, we performed Oct1 Western blotting using primary human metastatic pleural effusion breast carcinoma cells [37]. A variety of subtypes were tested (see figure legend). Unexpectedly, Oct1 protein expression was highly variable. Samples naturally partitioned into Oct1-high and -low categories (e.g., Figure 2B). We then determined whether Oct1 levels correlated with cancer–initiating cell frequency using CD24/44 as a measure of mammary tumor-initiating cells [6]. Pleural effusions with low Oct1 protein displayed low frequencies of CD24^LO^CD44^HI^Lin^NEG^ cells, whereas those with high Oct1 expression displayed a greater proportion. Examples from each category are shown in Figure 2C. Quantification from 15 samples is shown in Figure 2D. The observed differences were significant (*P* = 0.059). For the samples shown in Figure 2A, Oct1 mRNA levels were modulated in a similar manner to protein, however for other tested samples, changes in Oct1 message levels were insignificant (not shown). Performing the same analysis comparing CD24/44 with Oct1 mRNA yielded an insignificant *P*-value (Figure 2E). These results suggest that a combination of transcriptional/RNA regulation, but mostly regulation at the level of protein production or stability, underlies Oct1 variation in human breast cancer tissue.

### Oct1 controls stem cell markers in multiple tumor cell lines

**Figure 3:**
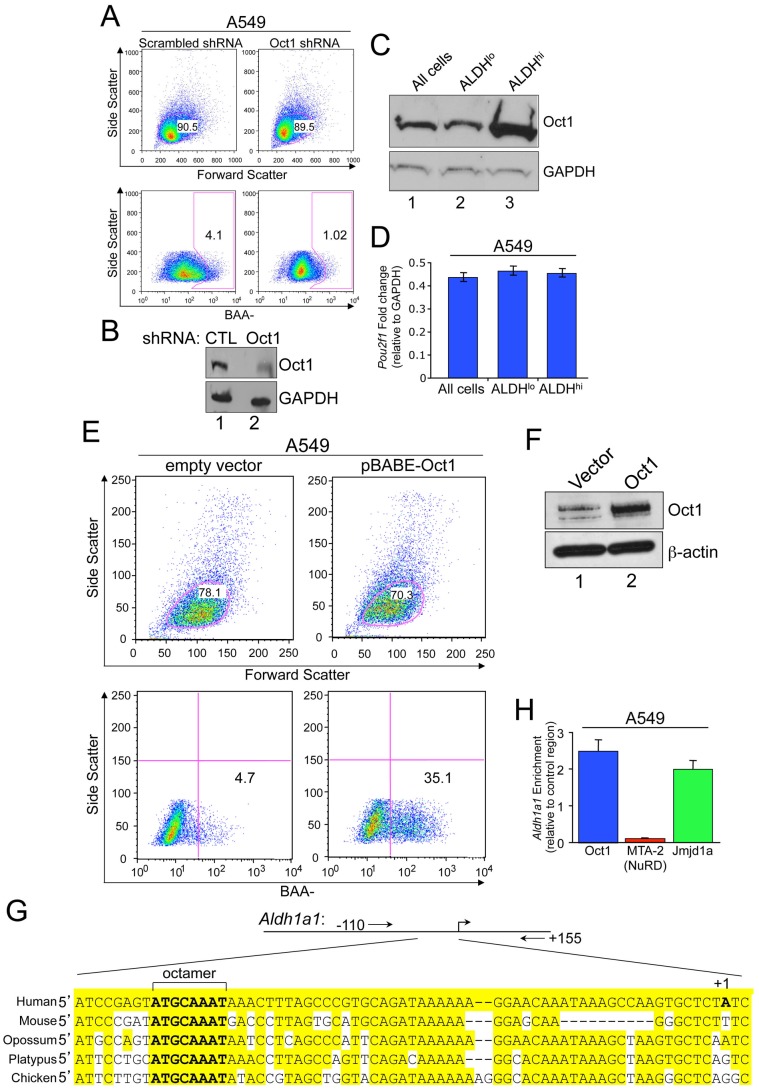
Oct1 controls the Aldefluor^HI^ population in human tumor cell lines. A. Aldefluor staining profile of A549 cells infected with a doxycycline-inducible lentiviral shRNA. MFI was 169.0 for scrambled and 91.2 for Oct1-specific shRNA. A549 cells were infected with control or Oct1-specific lentiviral particles (Santa Cruz), selected with puromycin, and subjected to analysis after 48 hr. B. Efficacy of the A549 knockdown as assessed by Western blotting using anti-Oct1 antibodies and an anti-GAPDH loading control. C. Oct1 Western blot of unsorted normal A549 cells cultured under normal conditions, and sorted Aldefluor^HI^ and Aldefluor^LO^ populations is shown. GAPDH is used as a loading control. D. The same sorted cells or unsorted cells were subjected to qRT-PCR to determine Oct1 mRNA levels. Levels are show relative to GAPDH. Error bars depict standard deviations. E. Oct1 was ectopically expressed in A549 cells using a retrovirus (pBabe-Oct1) or empty vector. The mixed population of cells was subjected to selection with puromycin, and ALDH activity determined. F. Western blot using anti-Oct1 antibodies of the same cells shown in (E). ß-actin is shown as a loading control. G. Alignment of the *Aldh1a1* promoter regions in several example vertebrate species. The conserved perfect octamer sequence centered at approximately −55 bp relative to the transcription start site is highlighted. Alignments were generated using a Clustal W-based algorithm within the Vector NTI software package (Invitrogen). Positions of PCR primer pairs for ChIP amplification are also shown. H. Quantification of ChIP enrichment using A549 cells and specific antibodies directed against Oct1, Mta2 (a component of the NuRD complex) and Jmjd1a. The PCR primer pair spanned the human *Aldh1a1* octamer site. ChIP enrichment was quantified relative to isotype control anti-C/EBPß antibodies and relative to a control region as described in the methods section. Values are the average of four independent experiments. Error bars represent standard deviations.

We used human epithelial tumor cell lines in which we could manipulate Oct1 levels by RNAi and retroviral overexpression. High ALDH activity, as measured by Aldefluor, is a marker of CSCs and tumor cell line populations with stem-like properties [9], [36]. We evaluated ALDH activity following Oct1 RNAi in A549 lung alveolar adenocarcinoma cells infected with pools of lentiviruses expressing scrambled or Oct1-specific shRNAs. Oct1-specific RNAi reduced activity in the main population by less than two-fold as measured by mean fluorescence. However, Oct1-specific RNAi more significantly impacted the number of ALDH^HI^ cells such that the Aldefluor^HI^ “tail” collapsed into a more symmetric distribution after Oct1-specific shRNA expression (Figure 3A). Effective RNAi was confirmed by Western blot (Figure 3B). We also studied ALDH activity in two breast cancer cell lines, MDA-MB-231 and MCF-7, using transiently transfected siRNA pools. Oct1 RNAi again minimally affected activity in the main population while significantly reducing the number of ALDH^HI^ cells (Figure S4). Therefore, in three tumor cell lines Oct1 ablation reduces ALDH activity most significantly in ALDH^HI^ cells.

Because an increase in Oct1 protein levels in stem-like Aldefluor^HI^ cells may underlie the selective effects of Oct1 ablation, we used fluorescence-activated cell sorting (FACS) to isolate normal A549 cells on the basis of Aldefluor activity and compared endogenous Oct1 protein levels. Oct1 levels were significantly increased in the Aldefluor^HI^ population relative to unsorted cells (Figure 3C). In contrast, no difference in *Oct1* (*Pou2f1*) mRNA was observed (Figure 3D). If elevated Oct1 protein is responsible for conferring an Aldefluor^HI^ phenotype, elevation of Oct1 protein levels should increase the population of Aldefluor^HI^ cells. A549 cells were infected with retroviruses encoding human Oct1 or empty vector controls. Oct1 overexpression did not grossly effect cells as measured by forward/side scatter (Figure 3E, top panels) or cell growth or viability [21], but did increase the proportion of Aldefluor^HI^ cells (Figure 3E, bottom panels). We confirmed Oct1 overexpression by Western blot (Figure 3F). These findings indicate that Oct1 controls the setpoint of Aldefluor^HI^ vs. Aldefluor^LO^ A549 cells.

An Oct1 binding site has been identified in the *Aldh1a1* immediate promoter region [38]. This site is highly conserved (Figure 3G). We conducted ChIP using normal A549 cells and the *Aldh1a1* promoter-proximal region to confirm Oct1 binding. A robust signal was observed using anti-Oct1 antibodies relative to an intergenic region and to an isotype control antibody (Figure 3H). Oct1 has been associated with two transcription cofactors, NuRD (in a negative regulatory capacity) and Jmjd1a (in a positive capacity), in different conditions [20]. ChIP using anti-Jmjd1a or anti-NuRD (Mta2) antibodies resulted in strong enrichment of Jmjd1a but not NuRD (Figure 3H), consistent with Oct1 mediating an activation function at *Aldh1a1* in A549 cells.

**Figure 4:**
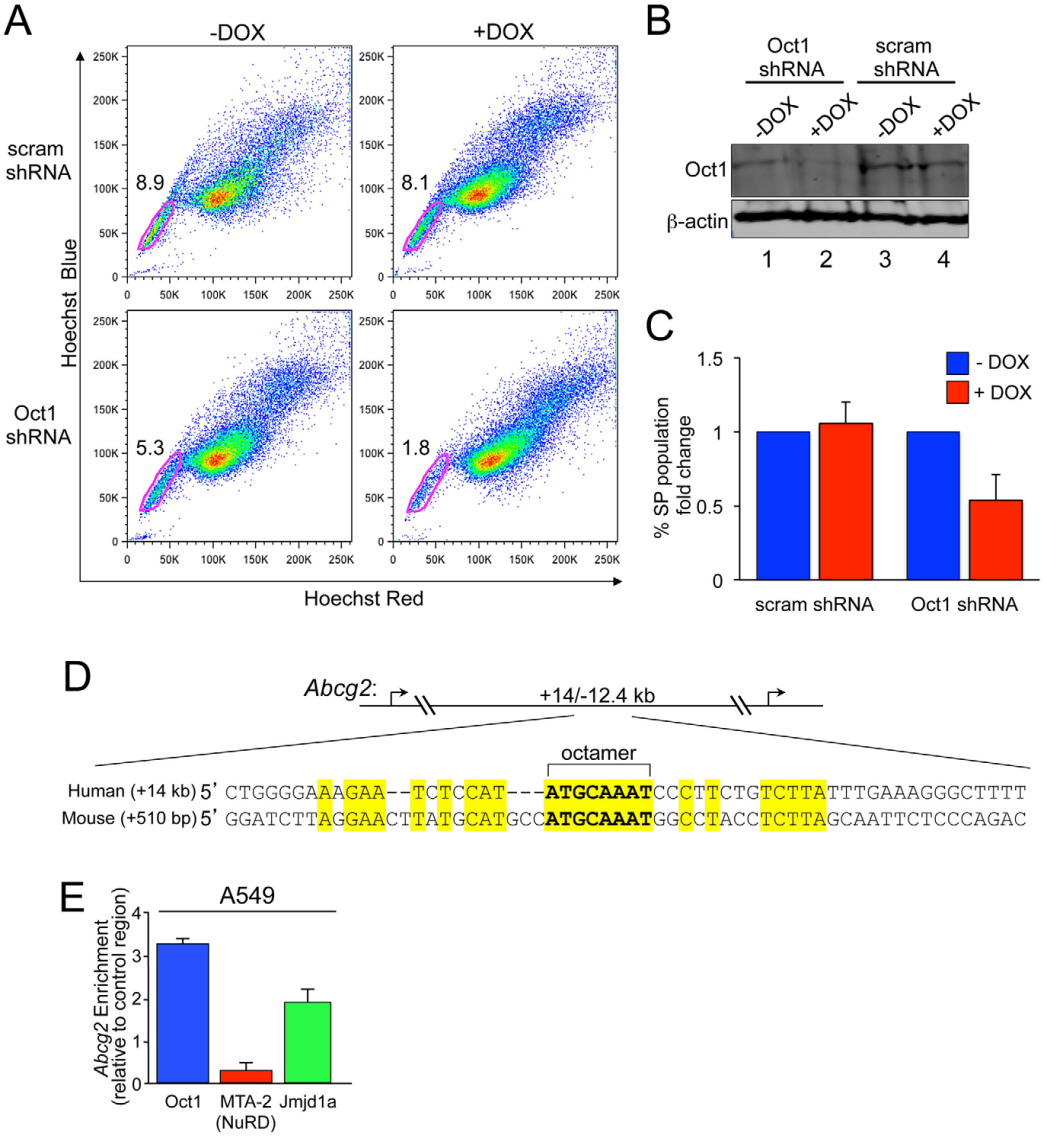
Oct1 RNAi diminishes the side population in A549 cells. A. SP assay of luciferase-positive A549 cells carrying an inducible scrambled or Oct1-specific shRNA [21]. Cells were treated with doxycycline for 4 days. Side population assays were conducted as described in the methods section. B. Western blot showing Oct1 protein levels in the two cell lines from (A) with and without 4-day culture in doxycycline. ß-actin is shown as a loading control. C. Quantification of the changed in SP percentage from three independent trials. Error bars denote standard deviations. D. Alignment of *Abcg2* first intron regions in human and mouse. The perfect octamer sequence in both species is highlighted. Human *Abcg2* has two annotated transcription start sites, so the element is located at both +14 kb and −12.4 kb relative to the transcription start sites. E. Quantification of ChIP enrichment using A549 cells and specific antibodies directed against Oct1, Mta2 and Jmjd1a. The PCR primer pair spanned the human Oct1 binding site in *Abcg2*. ChIP enrichment was quantified relative to isotype control anti-C/EBPß antibodies and relative to a control region as described in the methods section. Values are the average of four independent experiments. Error bars represent standard deviations.

To further buttress these findings we studied an independent stem cell marker. Normal and cancer stem cells are frequently dye efflux^HI^ such that incubation with Hoechst results in a fraction of cells (the side population, SP) that can be identified by low fluorescence [39]–[41]. Adenosine triphosphate (ATP)-binding cassette (ABC) multidrug transporters mediate this activity and contribute to the relative resistance to cytotoxic compounds associated with a stem cell phenotype [39], [42]. A549 cells contain a robust SP enriched in tumor-initiating cells [43]. To determine whether stable Oct1 knockdown selectively alters the SP, we used a previously established A549 inducible shRNA system [21]. A separate A549 clone inducibly expressing scrambled shRNAs was also used. Cells were stained with Hoechst Red, Hoechst Blue and propidium iodide. Dead cells, which were gated out, did not change significantly in the Oct1 depleted condition (not shown). Although the percentage of cells in the SP varied three-fold from experiment to experiment (and between A549 clones), induction of Oct1 shRNA by the addition of doxycycline uniformly and significantly reduced the SP, while minimally affecting the main population (Figure 4A, bottom panels). In contrast, little effect was observed upon doxycycline treatment of cells stably transduced with scrambled shRNAs (top panels). As expected, the SP was also reduced using the efflux transport inhibitor verapamil (not shown). Oct1 knockdown under these conditions was robust (Figure 4B). Averaged data from three independent experiments is shown in Figure 4C. These data show that Oct1 RNAi specifically decreases the SP in A549 cells.

ABC transporter G2 (ABCG2, also known as Bcrp1) regulates dye efflux activity, including in A549 cells [43], and stem cell chemoresistance [42]. We identified a consensus Oct1 binding element in the human *Abcg2* first intron (Figure 4D). ENCODE consortium data [44] indicates that the related Oct4 transcription factor interacts with this region in human ES cells. There is also a perfect octamer in the mouse *Abcg2* first intron (Figure 4D). FASTA alignment of the two sequences indicated that the homology is largely limited to the octamer element. ChIP indicated that Oct1 binds the *Abcg2* octamer element-containing region in A549 cells (Figure 4E). As with *Aldh1a1*, strong enrichment of Jmjd1a but not NuRD was observed (Figure 4E), consistent with Oct1 mediating an activation function at *Abcg2*.

Previous work identified an oxidative stress response mechanism in which Oct1 phosphorylation alters DNA binding specificity, causing induced Oct1 binding to DNA binding sites more complex than the canonical octamer element [19]. One such site is known as the MORE (More palindromic Octamer Related Element). Using H_2_O_2_-treated HeLa cells and ChIPseq, induced Oct1 binding was observed at MORE-containing targets such as *Hmgb3*, *Blcap*, *Rras* and *Rras2*. We identified another MORE sequence in the ABC transporter *Abcb1*, at position +250, and strong Oct1 ChIP enrichment at *Abcb1* in HeLa and A549 cells exposed to 1 mM H_2_O_2_ (Figure S5). Another transporter, *Abcb4*, is adjacent to *Abcb1* on human chromosome 7. ChIPseq previously identified inducible Oct1 binding to *Abcb4* following H_2_O_2_ exposure [19]. Inspection of this region revealed a MORE (not shown). We confirmed inducible binding in A549 cells (Figure S5).

### Oct1 controls tumor engraftment frequency

**Figure 5:**
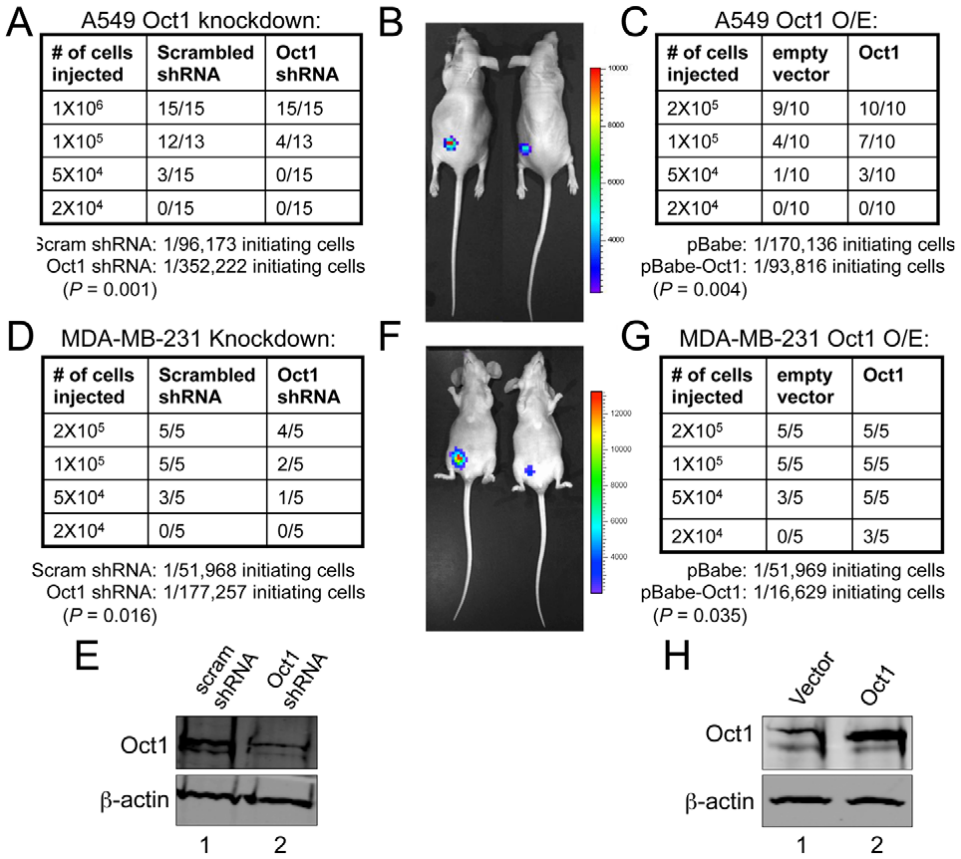
Oct1 controls tumor initiation frequency in A549 and MDA-MB-231 cells. A. Numbers of nude mice engrafted in the left flank with A549 cells expressing the same Oct1-specific shRNA as shown in Figure 4. Scrambled shRNAs in the contralateral flank were used as controls. The fraction of mice successfully engrafted is shown in tabular format. For the Oct1-specific and control shRNA lines, calculation of TIC frequency is shown at bottom. B. Images of engrafted tumors from animals receiving 1×10^5^ cells. Left flank: scrambled shRNA. Right flank: Oct1-specific shRNA. C. Similar to (A), except human Oct1 was over-expressed in luciferase-expressing A549 cells using retroviral gene transduction. D. 4^th^ inguinal mammary fat pads of nude mice were engrafted with the indicated number of luciferase-expressing MDA-MB-231 cells expressing scrambled or Oct1-specific shRNAs. The fraction of engrafted animals is shown in tabular format. E. Western blot showing efficacy of lentiviral knockdown. ß-actin is shown as a loading control. F. Images of engrafted tumors from animals receiving 2×10^5^ cells. Ventral view is shown. Right side: scrambled shRNAs. Left side: Oct1-specific shRNAs. G. Similar to (D) except human Oct1 was overexpressed using retroviral gene transduction. H. Western blot showing degree of Oct1 overexpression in cells used in (G). ß-actin is shown as a loading control.

The above findings indicate that Oct1 controls multiple markers and activities associated with stem cells, but do not address whether Oct1 controls the stem cell phenotype itself. We previously showed that stable Oct1 RNAi in luciferase-expressing A549 cells reduces tumorigenicity in xenograft assays without affecting growth rates in culture [21]. In these experiments, 2×10^6^ cells were transplanted and tumor mass was partially reduced (by approximately 60%). We hypothesized that differences in tumor initiating frequency underlie this effect. We injected reduced numbers of cells harboring scrambled or Oct1-specific shRNAs into opposite flanks of nude mice. Cells were pre-treated with doxycycline for 48 hr and injected into immunocompromised mice maintained on doxycycline. Using 1×10^6^ cells, both the scrambled and Oct1-specific shRNA-expressing cells engrafted 15/15 recipient mice. A further tenfold reduction resulted in 12/13 mice engrafted using cells expressing scrambled shRNAs while cells expressing Oct1-specific shRNAs engrafted 4/13 mice in the contralateral flank (Figure 5A, 5B). The remaining six mice showed no evidence of engraftment as assessed by visual inspection, palpation or bioluminescence. 50,000 cells engrafted poorly (4/15) using scrambled shRNAs and not at all with Oct1-specific shRNAs. Using even fewer cells, 0/15 mice engrafted regardless of Oct1 status (Figure 5B). These findings allowed us to calculate that Oct1 shRNA reduces the frequency of initiating cells from ∼1/96,000 to ∼1/350,000 (Figure 5A). We also over-expressed Oct1 in luciferase-expressing A549 cells. In this case no pre-treatment took place and the mice were not administered doxycycline. Oct1 overexpression was moderate (Figure 3F). This level of Oct1 expression leads to a >2-fold increase in TIC frequency (Figure 5C). Similar results were obtained with MDA-MB-231 human breast adenocarcinoma cells and mammary fat pad engraftment. Using Oct1 lentiviral knockdown, TIC frequency shifted downwards from ∼1/50,000 to ∼1/175,000 (Figure 5D–5F). Oct1 overexpression increased TIC frequency ∼3-fold from ∼1/50,000 to ∼1/17,000 (Figure 5G–5H).

### Oct1 regulates hematopoietic transplantation potential

Germline Oct1 deletion results in early embryonic lethality due to defects in extra-embryonic lineages, in particular trophoblast stem cells [45]. A slightly less severe Oct1 deficient allele dies over a wider developmental window beginning at mid-gestation (E11.5) and exhibits pale fetal liver, reduced ß-globin gene expression and reduced Ter119-positive cells [46]. Although these phenotypes are consistent with impaired hematopoietic stem cell (HSC) function, using this allele it was found that Oct1 deficient fetal livers reconstitute long-term B and T lymphopoiesis in adult recipients [47] and erythropoiesis in lethally irradiated hosts (not shown). To more carefully assess a cell-intrinsic role of Oct1 in hematopoiesis, we performed serial transplants, and competitive transplants using congenic markers.

**Figure 6:**
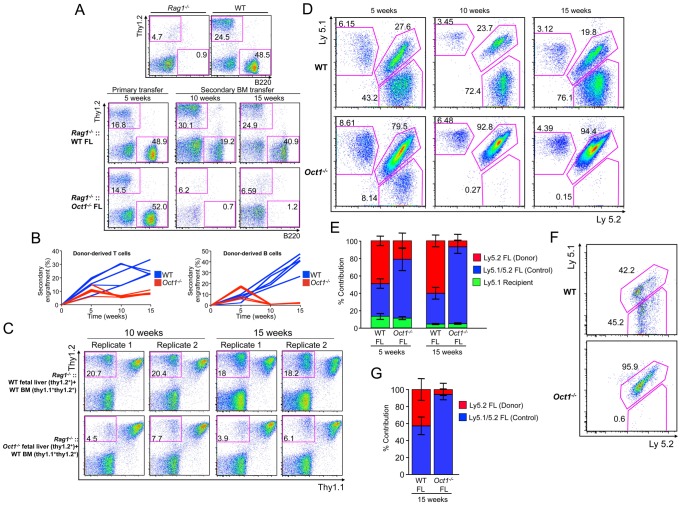
Loss of Oct1 interferes with hematopoietic engraftment. A. Peripheral blood leukocyte B and T cell profiles from primary and secondary animals transplanted with Oct1 deficient and WT littermate control fetal liver (FL). Cells were stained with anti-Thy1.2 and anti-B220 antibodies to reveal T and B cells. Control *Rag1^−/−^* and WT animals are shown at the top for comparison. B. B and T cell repopulation is shown as a percentage of total cells for 5 independent experiments. C. Flow cytometry plots showing degree of T cell reconstitution in peripheral blood leukocytes in four *Rag1^−/−^* recipient animals. For each animal, WT (top panels) or littermate Oct1 deficient (bottom panels) fetal livers were mixed with Thy1.1^+^/Thy1.2^+^ WT bone marrow (BM) and injected retro-orbitally. Peripheral blood repopulation was analyzed at 10 and 15 weeks. D. The same peripheral blood analysis as in (C) was performed using Ly5.1/2 instead of Thy1.1/2 as a congenic marker. A single mouse for each condition is shown. E. Averages of 4 WT and 4 Oct1 deficient competitive repopulations as performed in (D) are shown. Peripheral blood was analyzed at 5 and 15 weeks. Error bars depict +/− standard deviation. F. Bone marrow from the mice in (D) were gated on LSK and analyzed for Ly5 expression. G. Averages of the 4 WT and 4 Oct1 deficient competitive repopulations at the level of LSK bone marrow precursors.

In primary transplants Oct1 deficient fetal liver engrafted sublethally irradiated *Rag1^−/−^* primary recipients as evidenced by the presence of B220^+^ and Thy1.2^+^ Oct1 deficient donor B and T cells in peripheral blood (Figure 6A, primary transfer), consistent with prior findings in which engraftment is stable beyond 16 weeks [47]. Wild-type (WT) and *Oct1^−/−^* bone marrow from primary recipients also engrafted secondary recipients comparably at 5 weeks (not shown). However at later time points Oct1 deficient cells reproducibly showed nearly complete failure (Figure 6A, secondary transfer). Combined results from 5 independent trials are shown in Figure 6B.

Thy1.2^+^ WT or Oct1 deficient fetal liver cells were combined 1∶1 with Thy1.1^+^/Thy1.2^+^ WT bone marrow for competitive reconstitutions. Unlike WT littermate controls, *Oct1^−/−^* fetal liver cells engrafted poorly in the presence of WT bone marrow (Figure 6C). Similar results were obtained using the Ly5 marker, which can be used to detect a broader array of blood cells. Ly5.1^+^ C57BL/6 recipient mice were engrafted with WT or Oct1 deficient Ly5.2^+^ fetal liver cells combined 1∶1 with WT Ly5.1^+^/Ly5.2^+^ fetal liver cells. Again, Oct1 deficient cells were found in peripheral blood at 5 weeks, however large defects were observed, specifically using Oct1 deficient fetal liver, at 15 weeks (Figure 6D). Quantified results from four WT and four Oct1 deficient competitive situations is shown in Figure 6E.

To test whether the engraftment defect arises from a hematopoietic stem/progenitor cell defect, we analyzed Lin^NEG^Sca1^POS^c-kit^POS^ (LSK) bone marrow hematopoietic precursor cells in the above mice. Defects at least as robust as those seen in peripheral blood were observed in the Oct1 deficient fetal liver cell-derived bone marrow LSK compartment (two mice in Figure 6F, see Figure 6G for the whole cohort). These findings strongly suggest that Oct1 deficient LSK cells are less robust than their normal counterparts in competitive engraftment assays.

We also performed primary transplants using freshly isolated fetal liver cells from Oct1 deficient animals or wild-type littermate controls, or the same cells cultured for two days. Freshly isolated Oct1 deficient cells engrafted lethally irradiated recipient animals as before. Culture of the cells in media containing IL-3, IL-6 and SCF was sufficient to maintain engraftment potential in wild-type cells, but caused complete engraftment failure in Oct1 deficient cells (Figure S6). Oct1 regulates intracellular redox levels [21], [26]. Because culture with antioxidants had been found to correct a similar engraftment defect due to ATM deficiency [48], we incubated the fetal liver cells in cytokine-supplemented media in low-oxygen conditions or in the presence of N-acetylcysteine. Neither treatment restored engraftment potential (Figure S6).

## Discussion

Here we show that the Oct1 transcription factor (gene symbol *Pou2f1*, not to be confused with the organic cation transporter, *Oct1*) regulates the stem cell phenotype. The expression of Oct1 in multiple tissues, coupled with our findings in both epithelial and hematopoietic cells, indicates that it may control stem cell function in multiple compartments. Epithelial cells in colon and small intestine crypts show variegated Oct1 expression. The observation of variegated Oct1 expression is consistent with work in the developing eye [49]. Cells with high Oct1 protein expression also strongly express known stem cell markers, including Lgr5. These findings are consistent with a recent study [50], identifying Oct1 as one of a select group of factors whose protein but not mRNA levels are increased in isolated Lgr5-positive stem cells.

We examined four parameters of CSC phenotype and function: CD24/44 levels, dye efflux, ALDH activity and tumor initiating frequency in xenograft models. Elevated Oct1 expression correlates with ALDH1^HI^ cells in tumor sections, and with the contribution of CD24^LO^CD44^HI^ cells in breast tumor samples. Oct1 loss of function reduces dye efflux, ALDH activity and tumor initiating frequency in tumor cell lines. Oct1 protein levels are elevated in sorted ALDH1^HI^ populations, and forced Oct1 overexpression increases ALDH1^HI^ cells. Although these assays have their individual limitations [e.g.], [ 51,52], the common finding of an underlying role for Oct1 suggests that it is a controller of the CSC phenotype.

In contrast to Oct1 protein, the association between Oct1 mRNA levels and stem cell phenotypes is poor. This observation is consistent with findings that Oct1 target sites are highly enriched in the promoters of significantly up-regulated genes in lung and breast adenocarcinoma, leukemia, and myeloid leukemia stem cells without concomitant increases in Oct1 mRNA levels [53]–[57]. Elevated expression of Oct1 protein, but not mRNA, in stem cells may be due to increased rates protein synthesis, decreased rates of destruction, or both. Oct1 is known to be ubiquitinated [58] suggesting that regulated protein stability may be important, but the mechanism is unknown. Much of the increased Oct1 protein in stem cells appears to be cytoplasmic (Figure 1). The role of this cytoplasmic Oct1 is currently unknown, however Oct1 can be regulated at the level of nuclear/cytoplasmic localization [59], [60]. In addition, transcriptionally active Oct1 residing in the nucleus may be post-translationally modified in a way that alters its activity. Oct1 activity is regulated by cyclic AMP [60], cellular stress signals [61] and MAP kinase activity [20], however Oct1 post-translational modification states in stem cells and malignancy have not been carefully studied.

Oct1 may control stem cell phenotypes, in part, through its ability to regulate metabolism. Oct1 controls the expression of metabolic genes such as *Pcx* and *Pdk4* and dampens ROS levels [21], [26]. Reactive oxygen species (ROS) negatively modulate stem cell maintenance and self-renewal [62]. Stem cells are frequently characterized by glycolytic metabolic states and low ROS [13], [24]. Other relevant Oct1 targets include *Hmgb3* [61], with controls HSC function [63]. Here we show that Oct1 also associates with sites in the *Abcg2*, *Aldh1a1*, *Abcb1* and *Abcb4* target genes.

Loss of Oct1 reduces engraftment potential in competitive and serial hematopoietic repopulation assays, and compromises the LSK stem/progenitor cell compartment in competitive transplants. These results are consistent with fetal HSC deficiency, though because LSK is an impure population it is formally possible that Oct1 deficient HSCs engraft but function poorly. Bmi1 loss of function also results in hematopoietic failure [64]. As with Bmi1 [24], Oct1 is linked to mitochondrial function and the DNA damage response [21], [26], [61]. Hematopoietic defects are less readily apparent with Oct1 as compared to Bmi1. Both Dnmt1 deficiency and combined FOXO1/3/4 deficiency manifest milder hematopoietic defects similar to Oct1 [65], [66].

Previous studies identified embryonic pluripotency gene expression signatures in aggressive human breast carcinomas and in myeloid leukemia stem cells without observed Oct4 expression, suggesting a potential role for Oct4 paralogs [55], [56]. Oct1 and Oct4 share numerous common targets [19] and common modes of upstream regulation [61]. Therefore, an attractive model is that in those phenotypes common to ES cell pluripotency and somatic/cancer stem cells, Oct1 expressed at high levels mediates a subset of Oct4 functions. This may be particularly true if, in response to signals, Oct1 assumes additional or augmented functionalities.

## Materials and Methods

### Indirect immunofluorescence

For Figure 1A, frozen sections were fixed using 3.7% paraformaldehyde in phosphate buffered saline (PBS), and permeabilized using PBS with 0.05% Tween 20 (Sigma). Cells were stained with a mouse anti-Oct1 antibody (Milipore MAB5434), counter-stained using TO-PRO. Formalin-fixed paraffin-embedded human tissue microarrays (Imgenex) were used for images in Figure 1B and Figure 2A. Formalin-fixed paraffin-embedded mouse small intestine tissue blocks were sectioned and used for Figure 1D. Deparaffinization, hydration and antigen retrieval of human formalin-fixed paraffin-embedded tissue sections was performed as follows: Slides were incubated in a dry oven at 62°C for 1 hour, then dewaxed in xylene for 5×4 minutes, and hydrated in 100%, 95% and 75% ethanol for 2×3 minutes each. Slides were immersed in tap water for 5 minutes, then immersed in citrate buffer (0.01 M, pH 6.0), and microwaved on medium power for 5 min, then on low power for 5 min, and immersed in cold PBS. Non-malignant sections were blocked with 10% horse serum for 30 minutes in a humidified chamber, and incubated with rabbit anti-ALDH1a1 (Abcam ab52492) in 1% horse serum for 2 hours at room temperature. Sections were incubated with goat anti-rabbit Alexa488 (Invitrogen) for 1 hr at room temperature. Sections were blocked a second time with 1% BSA for 30 minutes at room temperature, then incubated with mouse anti-Oct1 (Millipore MAB5434) in 1% BSA overnight at 4°C. Sections were then incubated with goat anti-mouse Alexa568 (Invitrogen) for 1 hr at room temperature. Cells were mounted using media containing DAPI (Vector). For panels 1D and 2A, two mixed rabbit anti-Oct1 antibodies (Bethyl, A301-716A, A301-717A) were used. Panel 2A additionally used a mouse anti-ALDH1 antibody (Becton-Dickinson, BD 611194). Frozen mouse small intestine sections were used in panels 1C, 1E and 1F and were fixed in 1% paraformaldehyde then washed in PBS. Antigen retrieval was performed as published [67]. Mouse anti-Oct1 (Millipore MAB5434), goat anti-Lrig1 (R&D Systems AF3688) and rabbit anti-Lgr5 (Abgent AP2745d) were used with the M.O.M. kit (Vector labs) following the vendor protocol. Biotinylated mouse, goat and rabbit secondary antibodies (1∶50 dilution) were added, followed by streptavidin–horseradish peroxidase (Vector Vectastain Elite ABC kit). The signals were enhanced with the TSA kits NEL 741/744 (Perkin Elmer) according to the manufacturer's protocol, with fluorescein and Cy3 as the fluorophores.

### Cell culture

A549 and HeLa cells were maintained in DMEM (Invitrogen) supplemented with 10% serum (1∶1 calf∶fetal calf, Atlanta Biologicals), 6 mM L-glutamine/50 U/ml penicillin/50 µg/ml streptomycin (Invitrogen), and 50 µM ß-mercaptoethanol (Sigma). A549 cells expressing constitutive firefly luciferase and tet-inducible Oct1 shRNA were cultured and maintained as described previously [21]. MDA-MB-231 cells were engineered to express luciferase using a hygromycin-selectable cassette as for A549 cells [21]. Pleural effusions (PEs) from breast cancer patients were initially pelleted, and red blood cells were lysed by resuspending the pellet in ACK lysis buffer (150 mM NH_4_Cl, 10 mM KHCO_3_, 0.1 mM Na_2_EDTA) and incubating at room temperature for 10 min. The cells were re-pelleted and washed with DMEM/F-12 medium three times. During these washes, cancer cells were collected with rapid (1 minute) spins to enrich the pellet with tumor organoids. These cells were either immediately frozen in a solution of 10% DMSO/90% FBS, or maintained in DMEM/F12 1∶1 supplemented with 10 mM Hepes, 5% fetal bovine serum, 1 mg/ml bovine serum albumin, 1 µg/ml insulin with transferrin/selenium (Inivtrogen), 0.5 µg/ml hydrocortisone and 50 µg/ml gentamycin. All cells were maintained in 5% CO_2_ and air in a humidified 37°C incubator.

### Hematopoietic transplantations

Fetal liver cells were genotyped and transplanted, and *Rag1^−/−^* recipients analyzed as previously described [47]. Bone marrow cells (from femur and tibia) were depleted of red blood cells using ACK lysis buffer for 1 minute at room temperature. T cells were depleted using a biotinylated CD3 antibody (eBioscience) and anti-biotin microbeads (Miltenyi). For competitive transplants, 1.5×10^6^ Thy1.2^+^ WT or Oct1 deficient fetal liver cells were combined 1∶1 with Thy1.1^+^/Thy1.2^+^ WT bone marrow depleted of CD3^+^ T cells, and transplanted into *Rag1^−/−^* recipients via retro-orbital injection. For serial transplants, 1.5×10^6^ bone marrow cells from primary *Rag1^−/−^* recipient mice were used in the secondary transplant. Lethal radiation of WT C57BL/6 recipient animals was achieved with a split dose of 2×4.5 cGy, spaced 1 hr apart.

### Flow cytometry

For CD24/44 staining, PE cells were cultured overnight to allow the epithelial cells to adhere. Cells were then washed with PBS to deplete dead and hematopoietic cells. Epithelial cells were removed with trypsin-EDTA. Cells were stained with 7-AAD, anti-CD24, anti-CD44 and antibodies against lineage markers (CD2, CD3, CD10, CD16, CD18, CD31, CD64, CD140b) as published [6]. Non-viable and lineage-positive cells were gated out. CD24^LO^CD44^HI^ cell quantification was determined without prior knowledge of Oct1 expression levels. Aldehyde dehydrogenase activity was measured in cells as described [36] using the Aldefluor kit (Stem Cell Technologies) with 125 ng ALDH substrate and 100 mM DEAB (Sigma-Aldrich). Hoechst side population assays were performed as described [68], with the following modifications: dye incubation was performed in DMEM with 10% FBS, and the buffer for flow cytometry was PBS with 1 mM EDTA/0.5 mM EGTA. Cells were cultured in doxycycline (2 µg/ml) four days prior to analysis. Cells were co-stained with propidium iodide and dead cells were gated out from the analysis. Bone marrow LSK precursor cells were identified as described [69].

### qRT–PCR

RNA was isolated using Trizol (Invitrogen), followed by RNAeasy cleanup (Qiagen). cDNA was synthesized using Superscript III and random hexamers (Invitrogen). For *Pou2f1* RT-PCR, 100 ng of cDNA was used for quantitative RT-PCR using a LightCycler 480 (Roche). ΔCt values were determined by subtracting input DNA, and ΔΔCt was determined by subtracting the ΔCt value for control primers. The ΔΔCt were converted to fold change using the formula fold change = 2eΔΔCt and were averaged. Sequences for *Pou2f1* qRT-PCR were: *Pou2f1* forward, 5′ AAAAGAAATCAACCCACCAAGC; *Pou2f1* reverse, 3′ GCTAGTCACAAGGCTTGGTGT. Sequences for *Gapdh* qRT-PCR were: *Gapdh* forward, 5′ GGCCAAGGTCATCCATGACAA; *Gapdh* reverse, 3′AGGGGCCATCCACAGTCTTCT.

### RNAi

A549 cells were infected with lentiviral particles containing scrambled or pooled Oct1-specific shRNAs (Santa Cruz), and selected using puromycin. Inducible shRNA knockdown of Oct1 using A549 cells transduced with lentiviruses encoding three different shRNAs was described previously [21].

### Chromatin immunoprecipitation

A combination of two rabbit anti-Oct1 antibodies (Bethyl, A301-716A, A301-717A) were mixed and used for Oct1 immunoprecipitation. Anti-Jmjd1a and anti-NuRD (Mta2) antibodies were purchased from Abcam. ChIP conditions for the *Aldh1a1* and *Abcg2* regulatory regions were described previously [20]. Primer sequences for enrichment at *Aldh1a1* were: For, 5′ TTGAATCTTCAAATCGGTGAGTAGG; Rev, 5′ AAGTTTAAAGTCAAAGGCTTCCTGC. Primer sequences for enrichment at *Abcg2* were: For, 5′ ATGGCTTTACACTTTACCTGATCCC; Rev, 5′ TGAATGACATAGGTAGACCAGCACG. Intergenic primers were from an intergenic region of human chromosome 19 between the *Gadd45b* and *Lmnb2* loci. The sequences were: F2395, 5′ TTCTATGCCAAGCCCATTCTAGGTC; F2396 5′ GAGAGGCTCTGTCTGAGGTCACG. ChIP grade rabbit control IgG was purchased from Abcam (ab46540).

### Tumor xenograft

Luciferase-expressing A549 cells with inducible Oct1 shRNA knockdown [21] were cultured in the presence of doxycycline (2 µg/ml) for 48 hr and the indicated number injected subcutaneously into NCr nude mice (Taconic) provided with 2 mg/ml doxycycline in the drinking water two days prior and throughout the assay. Luciferase-expressing MDA-MB-231 cells were infected with Oct1-specific or control lentiviral knockdown constructs and implanted into the 4^th^ inguinal mammary fat pads of nude mice. For both cell lines, tumor engraftment was calculated at 8 weeks. Analyses were computed as previously described [70]. Briefly, analyses used the ‘statmod’ software package for the R computing environment (http://www.R-project.org). Tumor initiating cell frequencies were estimated using a complementary log-log generalized linear model. Two-sided 95% Wald confidence intervals were computed, except in the case of zero outgrowths, when one-sided 95% Clopper-Pearson intervals were used instead. The single-hit assumption was tested as recommended and was not rejected for any dilution series (*P*>0.05).

### Ethics statement

The study makes use of laboratory mouse models and primary human tissue. The latter were supplied commercially and from institutional samples. In those cases where institutional samples were used, institutional review board approval covering their use is on file at the University of Utah under the authors' names. Similarly, institutional animal care and use committee approval is present for all mouse procedures, and is on file under the authors' names. All procedures conformed to relevant regulatory standards.

## Supporting Information

Figure S1 Nuclear extracts were prepared from the Oct2-expressing human B cell line BJA-B or the Oct4 expressing human embryonic carcinoma cell line NCCIT. Western blots using mouse Oct2 (Calbiochem NA45), mouse Oct4 (Santa Cruz sc-5279) and rabbit Oct6 (Abcam ab31766) antibodies are shown, identifying a band of the appropriate molecular weight in each case. The same region was probed with the mouse Oct1 antibody (Milipore MAB5434). GAPDH is shown as a control.
(JPG)

Figure S2 Example immunohistochemistry image of formalin-fixed, paraffin-embedded normal human colon tissue. Longitudinal sections are shown. Sections were developed with peroxidase. Crypts are highlighted with dashed white lines. The same Oct1 antibody (Milipore MAB5434) was used. METHODS: IHC was performed using the Vectastain Standard ABC Kit Vector Laboratories) with ImmPACT DAB (oxidizable peroxidase substrate) as per manufacturer instructions.
(JPG)

Figure S3 Normal small intestine sections were stained as in Figure 2C, except A. Lgr5 antibodies were omitted, or B. Oct1 antibodies were omitted. For both (A) and (B), a more detailed image is shown below.
(JPG)

Figure S4 Oct1 RNAi diminishes the Aldefluor^Hi^ population in additional human tumor cell lines. A. Aldefluor staining profile of MB-MDA-231 cells. Transiently transfected siRNAs were used. MFI was 10.5 for scrambled and 8.2 for Oct1-specific siRNA. B. Efficacy of the MB-MDA-231 knockdown as assessed by Western blotting using anti-Oct1 antibodies and an anti-GAPDH loading control. C. and D. MCF-7 cells. Transiently transfected siRNAs were used. MFIs were 25.1 and 18.2. METHODS: MCF-7 human breast adenocarcinoma cells and MB-MDA-231 human breast adenocarcinoma cells were cultured identically to cells in the main manuscript, except that in the case of MCF-7 cells bovine insulin (Sigma, 0.01 mg/ml) was supplemented in the media. Transfection was accomplished using an Amaxa nucleofector. siRNAs pools were obtained from Ambion.
(JPG)

Figure S5 A. Identified MORE sequence in *Abcb1* first intron. Below is shown the alignment with 12 bona fide MORE sequences known to display inducible Oct1 binding following H_2_O_2_ exposure as measured by ChIP and ChIPseq (Kang et al., 2009, *Genes Dev.* 23:208). B. Oct1 ChIP enrichment at *Abcb1* in HeLa cells in comparison to a isotype control (red). Cells were treated with 1.0 mM H_2_O_2_ for 1 hr and ChIP enrichment monitored over a 4 hr window as per Kang et al (Kang et al., 2009, *Genes Dev.* 23:208). C. The same analysis with A549 cells and increasing doses of H_2_O_2_. D. Similar analysis with *Abcb4*. Cells were prepared at 1 hr. METHODS: ChIP was performed as published (Kang et al., 2009, *Genes Dev.* 23:208) with the following oligonucleotide PCR primers: Abcb1 for, 5′-GCTTCCATGTACC CCATTTCATAA; Abcb1 rev, 5′-AGCAGAAGTTTGTTGGCTGAGTG; Abcb4 for, 5′-GAGACATGTGTTTTGGAATGAGACAG; Abcb4 rev, 5′-CATCTGACGTATGTTCTCCATCTCC.
(JPG)

Figure S6 Fetal hematopoietic stem cell culture inhibits Oct1 deficient but not wild-type engraftment potential. Kaplan-Meier plots showing survival of mice repopulated with either wild-type or Oct1 deficient fetal liver cells. A. Survival of mice repopulated with freshly isolated fetal livers. B. Survival of mice repopulated with fetal livers cultured for 2 days in ambient oxygen. C. Survival of mice repopulated with fetal livers cultured for 2 days in 4% oxygen. D. Survival of mice repopulated with fetal liver cultured for 2 days in presence of 10 mM N-acetyl cysteine (Sigma). METHODS: 5×10^5^ fetal liver (E12.5) cells were injected into a lethally irradiated (900 Rad) mouse. Where culture conditions are used, fetal livers were cultured in RPMI supplemented with 20% fetal bovine serum, 20 ng/ml IL-3, 50 ng/ml IL-6, 50 ng/ml murine Stem Cell Factor (SCF, all cytokines from Fitzgerald), 100 U/ml penicillin, 100 mg/ml streptomycin and 2 mM L-Glutamine.
(JPG)
