# Supplementary figures and images for: Transcription Factor Oct1 Is a Somatic and Cancer Stem Cell Determinant
Source: PLoS Genet. 2012 Nov 8;8(11):e1003048. doi: 10.1371/journal.pgen.1003048 (PMC3493455; doi:10.1371/journal.pgen.1003048)

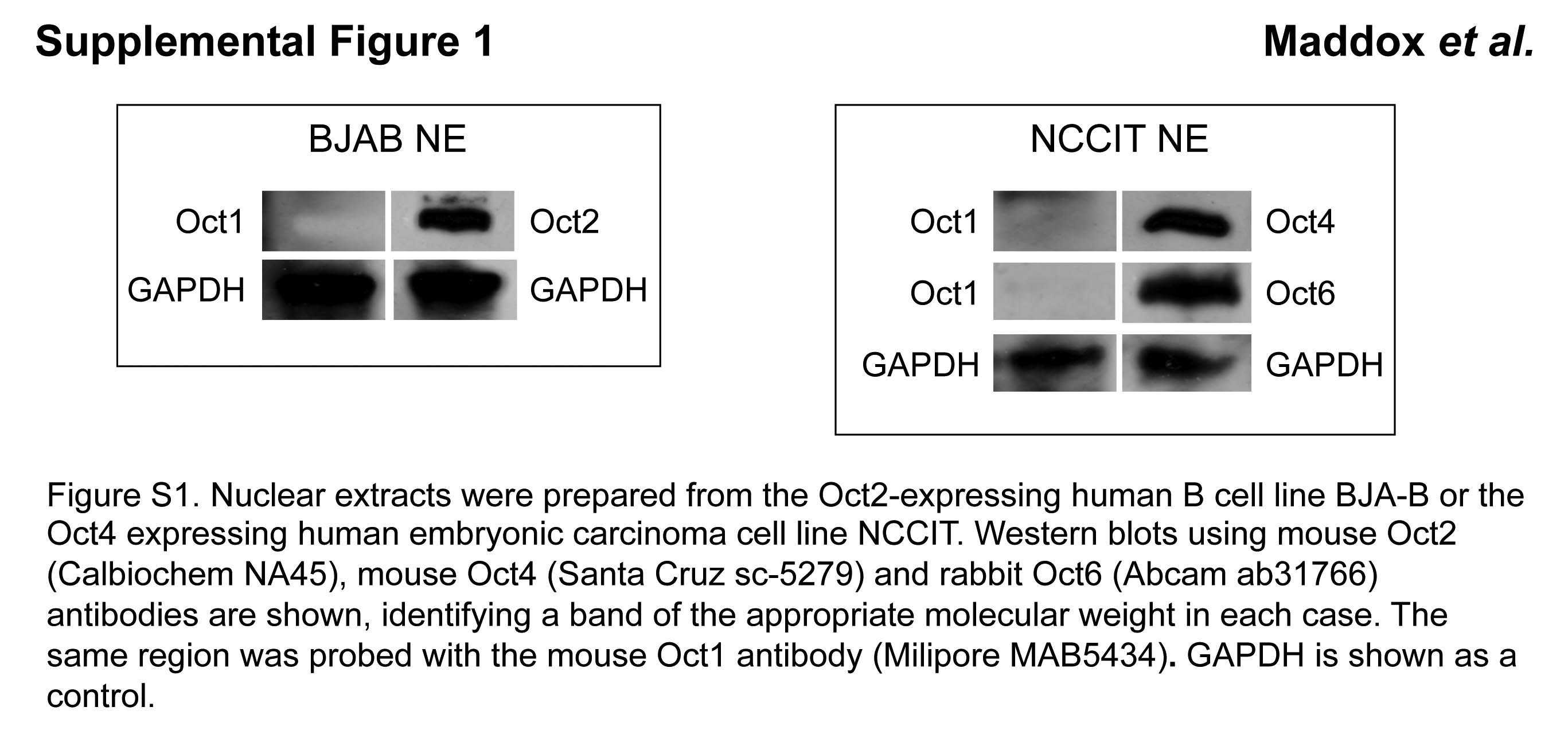

Supplement: Figure S1 — Nuclear extracts were prepared from the Oct2-expressing human B cell line BJA-B or the Oct4 expressing human embryonic carcinoma cell line NCCIT. Western blots using mouse Oct2 (Calbiochem NA45), mouse Oct4 (Santa Cruz sc-5279) and rabbit Oct6 (Abcam ab31766) antibodies are shown, identifying a band of the appropriate molecular weight in each case. The same region was probed with the mouse Oct1 antibody (Milipore MAB5434). GAPDH is shown as a control. (JPG) [file pgen.1003048.s001.jpg]

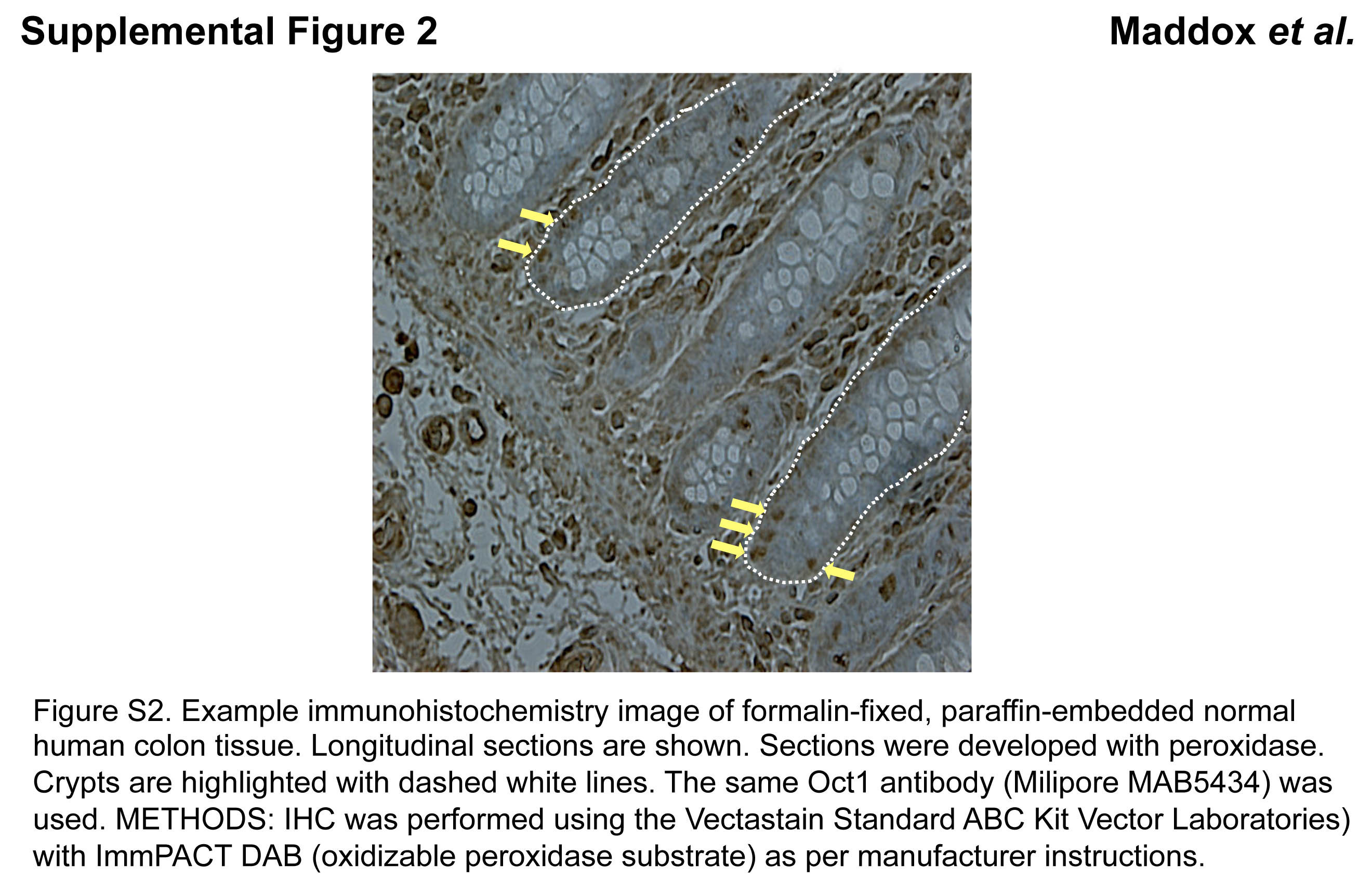

Supplement: Figure S2 — Example immunohistochemistry image of formalin-fixed, paraffin-embedded normal human colon tissue. Longitudinal sections are shown. Sections were developed with peroxidase. Crypts are highlighted with dashed white lines. The same Oct1 antibody (Milipore MAB5434) was used. METHODS: IHC was performed using the Vectastain Standard ABC Kit Vector Laboratories) with ImmPACT DAB (oxidizable peroxidase substrate) as per manufacturer instructions. (JPG) [file pgen.1003048.s002.jpg]

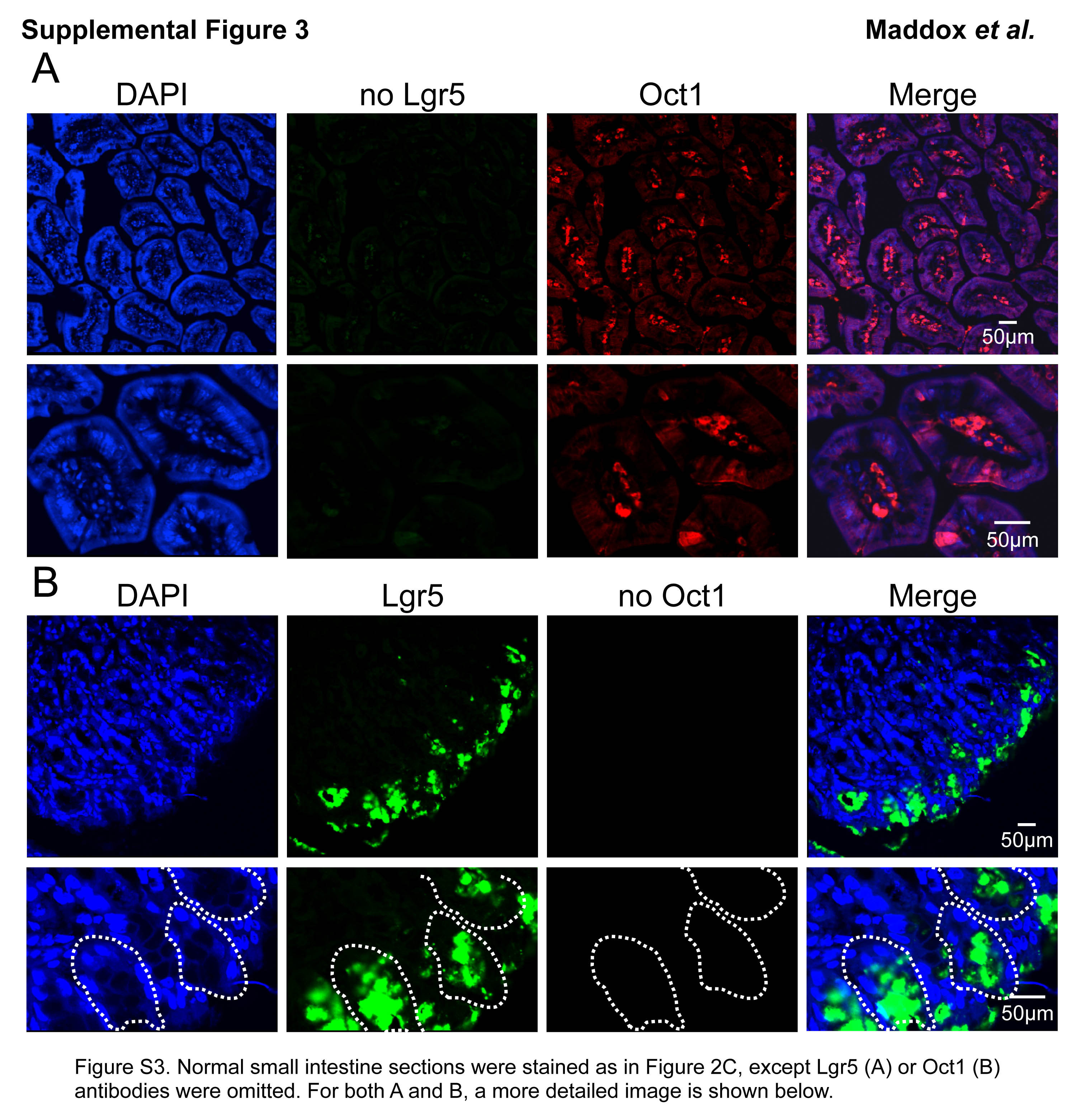

Supplement: Figure S3 — Normal small intestine sections were stained as in Figure 2C, except A. Lgr5 antibodies were omitted, or B. Oct1 antibodies were omitted. For both (A) and (B), a more detailed image is shown below. (JPG) [file pgen.1003048.s003.jpg]

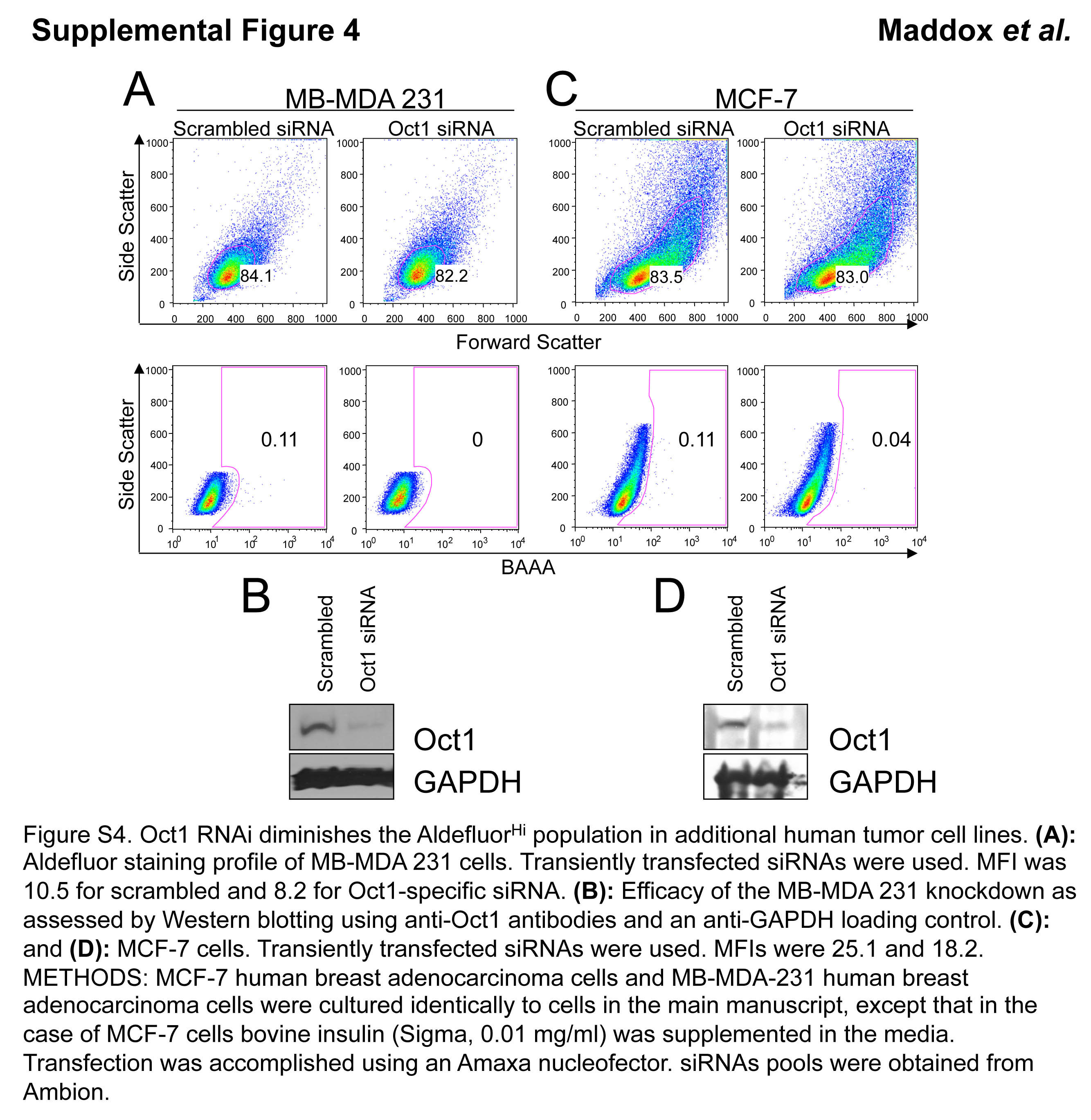

Supplement: Figure S4 — Oct1 RNAi diminishes the AldefluorHi population in additional human tumor cell lines. A. Aldefluor staining profile of MB-MDA-231 cells. Transiently transfected siRNAs were used. MFI was 10.5 for scrambled and 8.2 for Oct1-specific siRNA. B. Efficacy of the MB-MDA-231 knockdown as assessed by Western blotting using anti-Oct1 antibodies and an anti-GAPDH loading control. C. and D. MCF-7 cells. Transiently transfected siRNAs were used. MFIs were 25.1 and 18.2. METHODS: MCF-7 human breast adenocarcinoma cells and MB-MDA-231 human breast adenocarcinoma cells were cultured identically to cells in the main manuscript, except that in the case of MCF-7 cells bovine insulin (Sigma, 0.01 mg/ml) was supplemented in the media. Transfection was accomplished using an Amaxa nucleofector. siRNAs pools were obtained from Ambion. (JPG) [file pgen.1003048.s004.jpg]

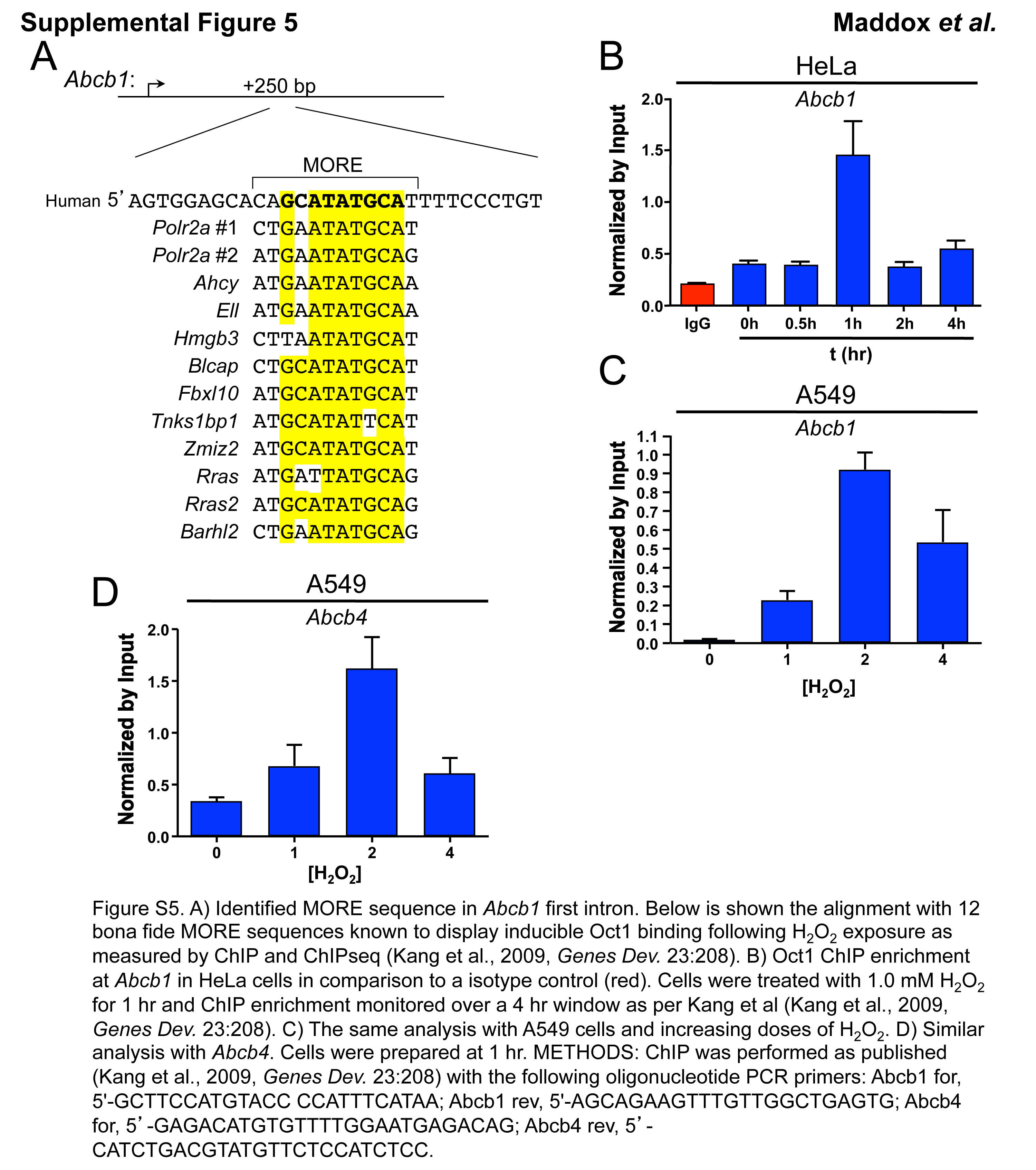

Supplement: Figure S5 — A. Identified MORE sequence in Abcb1 first intron. Below is shown the alignment with 12 bona fide MORE sequences known to display inducible Oct1 binding following H2O2 exposure as measured by ChIP and ChIPseq (Kang et al., 2009, Genes Dev. 23:208). B. Oct1 ChIP enrichment at Abcb1 in HeLa cells in comparison to a isotype control (red). Cells were treated with 1.0 mM H2O2 for 1 hr and ChIP enrichment monitored over a 4 hr window as per Kang et al (Kang et al., 2009, Genes Dev. 23:208). C. The same analysis with A549 cells and increasing doses of H2O2. D. Similar analysis with Abcb4. Cells were prepared at 1 hr. METHODS: ChIP was performed as published (Kang et al., 2009, Genes Dev. 23:208) with the following oligonucleotide PCR primers: Abcb1 for, 5′-GCTTCCATGTACC CCATTTCATAA; Abcb1 rev, 5′-AGCAGAAGTTTGTTGGCTGAGTG; Abcb4 for, 5′-GAGACATGTGTTTTGGAATGAGACAG; Abcb4 rev, 5′-CATCTGACGTATGTTCTCCATCTCC. (JPG) [file pgen.1003048.s005.jpg]

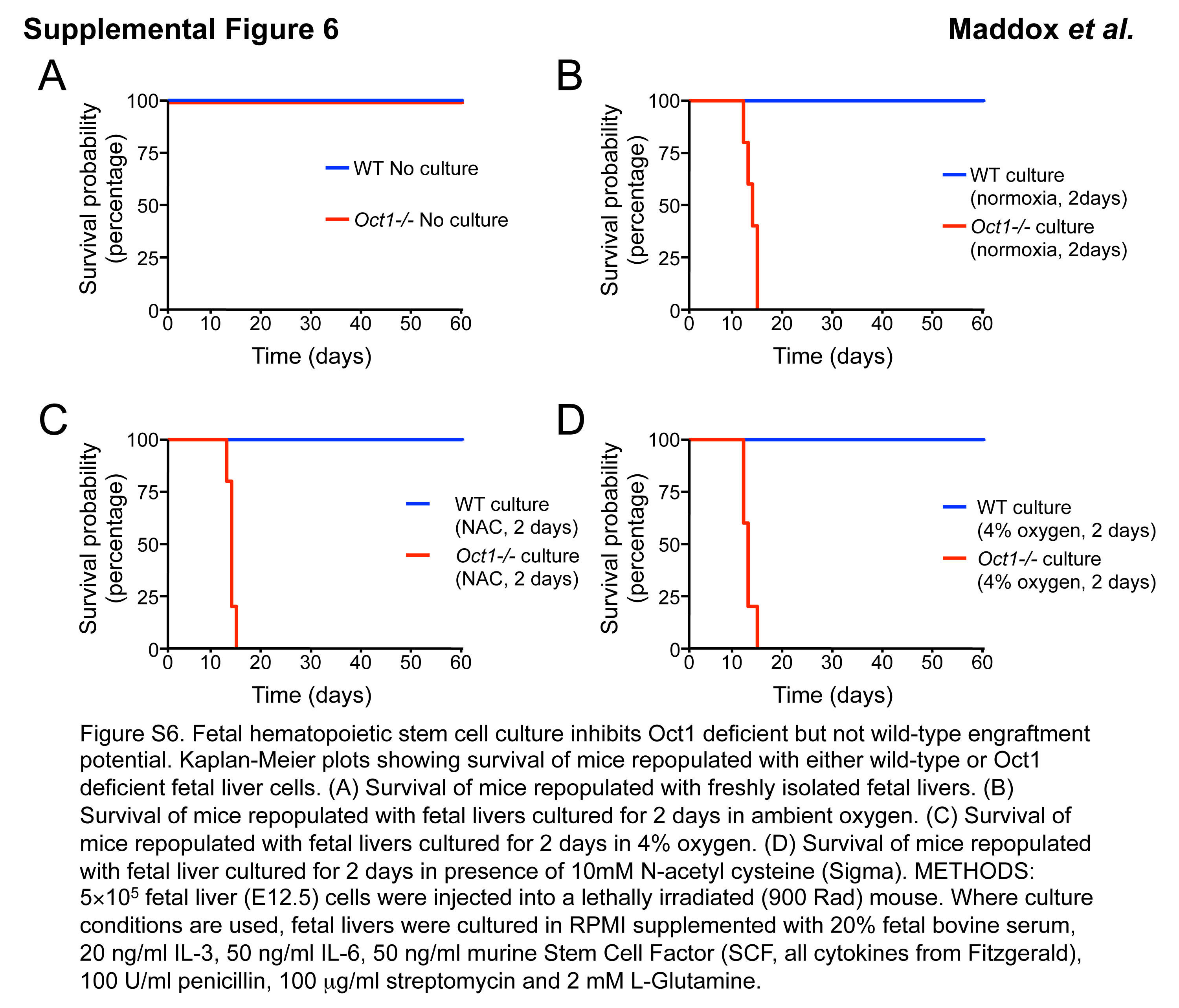

Supplement: Figure S6 — Fetal hematopoietic stem cell culture inhibits Oct1 deficient but not wild-type engraftment potential. Kaplan-Meier plots showing survival of mice repopulated with either wild-type or Oct1 deficient fetal liver cells. A. Survival of mice repopulated with freshly isolated fetal livers. B. Survival of mice repopulated with fetal livers cultured for 2 days in ambient oxygen. C. Survival of mice repopulated with fetal livers cultured for 2 days in 4% oxygen. D. Survival of mice repopulated with fetal liver cultured for 2 days in presence of 10 mM N-acetyl cysteine (Sigma). METHODS: 5×10^5^ fetal liver (E12.5) cells were injected into a lethally irradiated (900 Rad) mouse. Where culture conditions are used, fetal livers were cultured in RPMI supplemented with 20% fetal bovine serum, 20 ng/ml IL-3, 50 ng/ml IL-6, 50 ng/ml murine Stem Cell Factor (SCF, all cytokines from Fitzgerald), 100 U/ml penicillin, 100 mg/ml streptomycin and 2 mM L-Glutamine. (JPG) [file pgen.1003048.s006.jpg]
